# Combined Effects of Prenatal Exposures to Environmental Chemicals on Birth Weight

**DOI:** 10.3390/ijerph13050495

**Published:** 2016-05-12

**Authors:** Eva Govarts, Sylvie Remy, Liesbeth Bruckers, Elly Den Hond, Isabelle Sioen, Vera Nelen, Willy Baeyens, Tim S Nawrot, Ilse Loots, Nick Van Larebeke, Greet Schoeters

**Affiliations:** 1 Environmental Risk and Health, Flemish Institute for Technological Research (VITO), 2400 Mol, Belgium; elly.denhond@wiv-isp.be (E.D.H.); greet.schoeters@vito.be (G.S.); 2 Department of Epidemiology and Social Medicine, University of Antwerp, 2610 Wilrijk, Belgium; sylvie.remy@uantwerpen.be; 3 Interuniversity Institute for Biostatistics and Statistical Bioinformatics, Hasselt University, 3590 Diepenbeek, Belgium; liesbeth.bruckers@uhasselt.be; 4 Department of Public Health, Ghent University, Ghent, Belgium; FWO Research Foundation, 1000 Brussels, Belgium; isabelle.sioen@UGent.be; 5 Department of Health, Provincial Institute for Hygiene, 2000 Antwerp, Belgium; Vera.NELEN@provincieantwerpen.be; 6 Department of Analytical, Environmental and Geochemistry (AEGC), Vrije Universiteit Brussel, 1040 Brussels, Belgium; wbaeyens@vub.ac.be (W.B.); nicolas.vanlarebeke@ugent.be (N.V.L.); 7 Centre for Environmental Sciences, Hasselt University, 3590 Diepenbeek, Belgium; tim.nawrot@uhasselt.be; 8 Department of Public Health & Primary Care, Leuven University, 3000 Leuven, Belgium; 9 Department Sociology, Faculty of Political and Social Sciences, University of Antwerp, 2000 Antwerp, Belgium; ilse.loots@uantwerpen.be; 10 Department of Biomedical Sciences, University of Antwerp, 2610 Wilrijk, Belgium; 11 Department of Environmental Medicine, University of Southern Denmark, 5230 Odense, Denmark

**Keywords:** endocrine disruptors, mixtures, principal component analysis, regression analysis, birth outcome, epidemiology, biomonitoring, cord blood

## Abstract

Prenatal chemical exposure has been frequently associated with reduced fetal growth by single pollutant regression models although inconsistent results have been obtained. Our study estimated the effects of exposure to single pollutants and mixtures on birth weight in 248 mother-child pairs. Arsenic, copper, lead, manganese and thallium were measured in cord blood, cadmium in maternal blood, methylmercury in maternal hair, and five organochlorines, two perfluorinated compounds and diethylhexyl phthalate metabolites in cord plasma. Daily exposure to particulate matter was modeled and averaged over the duration of gestation. In single pollutant models, arsenic was significantly associated with reduced birth weight. The effect estimate increased when including cadmium, and mono-(2-ethyl-5-carboxypentyl) phthalate (MECPP) co-exposure. Combining exposures by principal component analysis generated an exposure factor loaded by cadmium and arsenic that was associated with reduced birth weight. MECPP induced gender specific effects. In girls, the effect estimate was doubled with co-exposure of thallium, PFOS, lead, cadmium, manganese, and mercury, while in boys, the mixture of MECPP with cadmium showed the strongest association with birth weight. In conclusion, birth weight was consistently inversely associated with exposure to pollutant mixtures. Chemicals not showing significant associations at single pollutant level contributed to stronger effects when analyzed as mixtures.

## 1. Introduction

Over the last decade, epidemiological studies have frequently shown that the intra uterine environment does not always sufficiently protect the fetus from environmental factors. Maternal stress, dietary factors, and exposure to environmental chemicals have a measurable influence on fetal growth and fetal development with consequences on birth outcome, child development and adult health [1,2,3]. Evidence is accumulating that early life exposures induce changes in fetal growth patterns. Altered fetal programming leads to non-adaptive postnatal responses that become manifest as excessive weight gain and insulin resistance [4]. These are risk factors for adult diabetes, cardiovascular diseases and certain cancers [5]. As overweight and obesity reach epidemic proportions, it is very important to identify the risk factors. 

Several xenobiotic chemicals are suspected obesogens because they have been associated with effects on fetal and/or postnatal growth in human birth cohorts and follow up studies. Most of the evidence points to organochlorines with endocrine properties such as polychlorinated biphenyls (PCBs), dichlorodiphenyldichloroethylene (DDE), hexachlorobenzene (HCB) or plasticizers such as phthalates, bisphenols and many more new chemicals that are present in consumer products are suspect [6,7]. Effects of particulate matter on birth weight have been described in cohorts often of more than ten thousands individuals [8,9]. Metals such as cadmium [10] are also suspected of having an impact on fetal growth based on experimental evidence from animal studies or mechanistic information from *in vitro* studies. Studies with perfluorinated compounds have so far yielded inconsistent results [11]. Meta analyses and recent reviews support the evidence for selected chemicals but individual studies give often conflicting results [12,13]. One of the reasons may be the presence of different pollutant mixtures in populations living in different environments and with different life styles.

Most studies associate individual pollutant exposure to health outcomes. However, this does not comply with real life situations as humans are exposed to hundreds of manmade chemicals during their lifetime. Even at the start of life, human biomonitoring shows that complex mixtures of xenobiotic chemicals are present in the prenatal environment [14]. Environmental health surveillance programs use human biomonitoring increasingly, as it accurately measures the internal pollutant load absorbed into the human body and integrates uptake by various exposure routes [15,16]. The progress made in analytical chemistry allows quantifying a large number of chemicals yielding a broad spectrum of chemical read outs from the same biological sample. The challenge is to identify the specific components of chemical mixtures that are most critical to the outcome of interest. Compounds that are correlated may be a proxy for each other in analyses while their biological action may be very different [17]. To address the critical chemical is pivotal for risk assessment and effective risk management. Another challenge is the potential interaction between the chemicals: they may counteract, act in synergy, add up or may have different targets. Mechanistic studies have demonstrated the existence of all these possibilities [18]. Interactions may be nonlinear and may vary in function of the dose [19]. Further complexities are the presence or absence of a chemical threshold for the effect and the different shapes of the dose-response curves of the individual chemicals which may be nonlinear for some chemicals [20]. 

Statistical analyses of multi-pollutant exposure are complex. Several approaches have been suggested to deal with health effects of complex exposures, among which most methods were applied to the field of air pollution [21,22]. Fewer epidemiological studies deal with multiple human exposures using biomonitoring data. These studies applied data reduction methods, e.g., by constructing principal components [23] or calculating and summing individual risk-scores for each of the exposure markers [24]. Other studies used non-parametric methods that do not use distribution assumptions or linearity [25], such as regression tree analysis. Other techniques were Bayesian modeling [26], elastic net regression [27], and performing regression by a step by step algorithm. 

In the Flemish human environmental health survey (FLEHS), human biomonitoring is used to assess xenobiotic exposure at the start of life in a sample of the Flemish population that is representative for geographical distribution and is weighed for population density [28]. Since Flanders is one of the most populated areas of Europe with dense traffic, important harbors and industry, an environmental health surveillance program is installed for monitoring environmental exposures and associated effects. The contaminants were selected based on prioritization criteria described in Schoeters *et al.* [28]. Thus far exposure response/effect associations have been studied by multiple regression analysis or structural equation analysis. In this study, we explored the hypothesis that a multiple exposure profile to study exposure-effect relations provides additional information on health effects as compared to studying the effect of pollutant exposure one by one. We investigated whether we can distinguish among the exposure biomarkers that are measured in cord blood samples and maternal blood, exposures or their combinations that have an effect on birth weight and whether the effect of the combinations is more pronounced than the effect of single exposure biomarkers. To evaluate the effect of multiple exposures simultaneously, we constructed principal components to reduce the number of exposure variables and propose a novel approach. The results are compared to the outcomes of single pollutant multiple regression analyses.

## 2. Experimental Section 

### 2.1. Study Population

Data from the FLEHS II mother-child cohort were used. The study protocol has been described elsewhere [29]. Briefly: 248 newborn-mother couples were recruited from the general population of the five provinces of Flanders between August 2008 and July 2009 using a multistage sampling procedure. The population consisted exclusively of uncomplicated liveborn singleton pregnancies. Participation criteria included living in Flanders for at least 10 years, ability to fill in a Dutch questionnaire, and giving birth in one out of ten randomly selected maternities. The distribution of participants over the different provinces was in accordance with the number of inhabitants in that province (status at 1 January 2006). 

The birth outcome of interest was birth weight. The information was obtained from the medical records of the maternities. Covariate/confounder data of individual health socio-demographic and life style characteristics, reproductive history and health were obtained from questionnaires. The characteristics of the study population are presented in Table 1. Written informed consent was provided by all mothers that participated in this study. The mothers also signed the informed consent on behalf of the children enrolled in the study. The study protocol was approved by the ethical committee of the University of Antwerp (Reference UA A08 09). 

### 2.2. Chemical Exposures

Our analysis included 15 chemicals that were measured in individual samples. Lead (Pb), manganese (Mn), copper (Cu), thallium (Tl) and arsenic (As) were measured in cord blood, cadmium (Cd) was measured in maternal whole blood samples collected after birth in the maternity. The metals were measured by high resolution inductively coupled plasma-mass spectrometry (HR-ICP-MS, Thermo Element II) after micro-wave acid digestion using HNO_3_ and H_2_O_2_ [30]. Persistent chlorinated compounds (polychlorinated biphenyl-138, -153 and -180 (PCB-138, PCB-153, and PCB-180, respectively) and dichlorodiphenyldichloroethylene (p,p’-DDE)), were measured in plasma cord samples by using solid-phase extraction and gas chromatography-electron capture negative ionization mass spectrometry [31,32]. CALUX (Chemical Activated Luciferase gene eXpression) assay is a bioassay used to measure the dioxin like compounds in cord plasma [33]. The serum concentration of the organochlorine pesticides and the PCBs were lipid-normalized in units of ng/g serum lipid. Perfluorinated compounds (perfluorooctane sulfonate (PFOS), perfluorooctanoic acid (PFOA)) in cord plasma samples were measured according to Midasch *et al.* [34]. Total mercury (Hg) and methylmercury (Hg-MM) were measured in maternal hair by combustion-atomic absorption spectrometry and by headspace injection-gas chromatography-atomic fluorescence spectrometry, respectively [35]. Hg-MM and not total Hg was used in data analysis. Mono-(2-ethyl-5-carboxypentyl) phthalate (MECPP), a metabolite of di(2-ethylhexyl) phthalate (DEHP), in cord plasma was measured after enzymatic deconjugation [36]. Other metabolites of DEHP were also measured, *i.e.*, mono(2-ethyl-5-hydroxyhexyl)phthalate (MEHHP), and mono(2-ethyl-5-oxohexyl)phthalate (MEOHP). However, these could only be detected in a minority of the samples and are therefore not included in the analysis. 

In Belgium, several air pollutants, including fine particulate matter (PM_2.5_), are continuously measured by a network of automatic monitoring sites [37]. To estimate air quality at locations where no measurements are made, the RIO model is used [38]. The RIO model interpolates the available measurements from permanent air quality monitoring stations, while taking into account information about land use [38,39]. RIO allows assessing a single average concentration per 4 × 4 km² grid cell. The model was used to calculate 24 h average concentrations of PM_2.5_ at the home address of individual participants. Daily concentrations were subsequently averaged over the whole duration of pregnancy.

### 2.3. Statistical Analysis

Data pre-treatment was applied to improve the robustness of the statistical methods, meet the assumptions of statistical tests or improve graphical visualization of the data. Exposure biomarkers with measurements below the limit of detection (LOD) or quantification (LOQ), depending on which cut-off the measuring laboratory applied, were imputed by half the value of the LOD or LOQ. All exposure biomarkers were ln transformed, as the distributions were skewed. Then, the biomarker data were normalized, *i.e.*, expressing each exposure biomarker as its Z-score, which was calculated by subtracting the mean and dividing by the standard deviation for each biomarker. This assured that the data are on a common scale that does not span orders of magnitude. Correlations between the Z-scores of the 16 exposures markers were assessed by calculating the Pearson correlation coefficients. Database management and statistical analyses were performed with SAS software version 9.3 (SAS Institute Inc., Cary, NC, USA) and Matlab (version 2014b, The Mathworks^®^, Natick, MA, USA). Statistical significance was defined as *p*-value < 0.05.

#### 2.3.1. Single Pollutant Regression Models

Linear regression models were used to quantify the effect on birth weight associated with an interquartile range (IQR) increase in Z-score of one pollutant. The exposure-response relations were adjusted for *a priori* fixed known confounders based on literature reviews, *i.e.*, gestational age, gender of the newborn and smoking during pregnancy. Other influencing factors (covariates) were included when significant at the 0.05 level in the multiple regression model. The following covariates were considered: maternal age, parity, stress/pressure during pregnancy, maternal educational level, smoking before pregnancy, alcohol use before/during pregnancy, maternal height, maternal prepregnancy body-mass index (BMI), equivalent income, infections/complications during pregnancy, use of folic acid during pregnancy and caesarean section. We evaluated sex and smoking status as potential effect modifiers as indicated by several previous studies [40,41,42,43,44,45,46]. Effect modification (interaction) was analyzed in models including main effects and cross-product terms. A *p*-value < 0.10 for the effect of the cross-product was taken as an indication for interaction. Informal diagnostic plots and formal tests were applied to check assumptions of normality (Kolmogorov–Smirnov test), constancy of variance (White’s General test [47]), independence (randomness), and linearity (lack of fit test [48]). Detection of outliers and influential data points was based on influence statistics (residuals, leverage). Regression models were fitted with and without these influential outliers. 

#### 2.3.2. Principal Component Regression 

Principal components regression (PCR) is a method to model a response variable when there are a large number of predictor variables (exposure biomarkers in this study), and those predictors are correlated or even collinear [49]. New predictor variables, known as principal components (PCs), are constructed as linear combinations of the original predictor variables. By the technique of principal component analysis (PCA), these principal components are created to explain the observed variability in the predictor variables. PCs represent variation in the original data set; the first PC represents the maximum amount of variation possible in one dimension, the second PC represents the maximum amount of the remaining variation in one dimension perpendicular to the first PC, and so on for all remaining PCs. PCA with varimax rotation was applied. PCA is a data reduction tool and has been used for identifying patterns of exposure from complex chemical mixtures [22,50,51,52,53,54,55]. The critical eigenvalue a component must display if that component is to be retained is specified as 1. PCA needs complete data for all the exposure variables and covariates. Since there were a lot of missing exposure measures depending on the biomarker, a sensitivity analysis was performed using the subset of exposure biomarkers with the highest sample size and in the same time with as many exposure biomarkers as possible. The resulting principal components were linked to birth weight by the same multiple linear regression models as described for the single pollutant models, and adjusted for confounders and statistically significant covariates.

#### 2.3.3. Exploring the Effect of Mixtures

**Figure 1:**
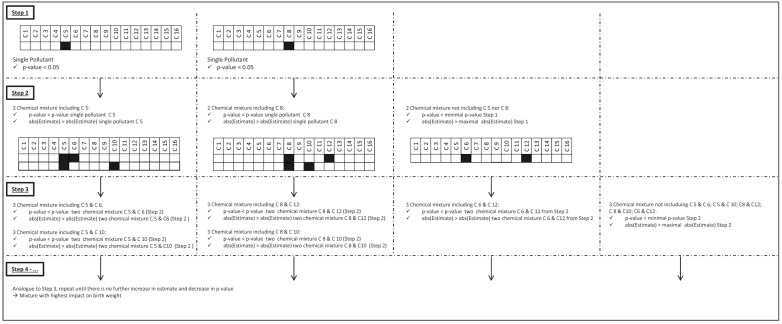
Mixture regression algorithm. Starting from single pollutant models, significant associations (*p*-value < 0.05) between exposure and birth weight were selected. Alternatively, when significant associations at single pollutant level were lacking, the single pollutant association with the highest rank (based on *p*-value) was selected. Next, the association of mixtures composed of N chemicals was evaluated by comparing to the association of N-1 chemicals. The criteria to identify models having a stronger association as compared to least complex mixtures were based on the strength of the estimate and *p*-value of the association between the average mixture z-score and birth weight.

Based on a dataset of 16 exposures, 65,519 different mixtures can be constituted consisting of 2 to 16 exposures. We quantified each possible mixture by averaging the Z-scores. Subsequently, linear regression models were used to quantify the effect on birth weight associated with an interquartile range (IQR) increase of a given mixture. As such, this approach was independent from the results obtained by PCA analysis. Pearson correlation was performed to decide whether pairs of exposure were highly correlated. Highly correlated variables were summed and considered as 1 exposure variable when deriving average Z-scores with other exposures. The exposure-response relations were adjusted for *a priori* fixed known confounders (gestational age, gender of the newborn and smoking during pregnancy) and for other influencing factors (covariates) that were determined as described in the paragraph above. Effect modification (interaction) by sex of the newborn and smoking status was considered when there was an indication for interaction (*p*-value < 0.10 for the effect of the cross-product) in the single pollutant regression models. We developed an algorithm to determine the least complex mixture that showed the highest association with birth weight (Figure 1). First, significant single pollutant models were identified (*p*-value < 0.05). Alternatively, when significant associations at single pollutant level were lacking, the single pollutant association with the highest rank (based on *p*-value) was selected. Next, the association of mixtures composed of N chemicals was evaluated by comparing to the association of N-1 chemicals. The criteria to identify models having a stronger association with birth weight as compared to less complex mixtures were based on the estimate and *p*-value of the association between the average mixture Z-score and birth weight as depicted in Figure 1. As our goal was to detect mixtures of pollutants with an increased estimated effect on birth weight, no selection criteria like likelihood ratio or mean square error were used to identify a ‘better’ model. This approach enables to identify associations with mixtures of different origin.

## 3. Results

Our analysis included 248 singleton newborns with data on birth weight. The characteristics of the population are presented in Table 1. Most mothers came to deliver their first child (39.9%), the majority was highly educated (60.1%), and 11.7% of the mothers smoked during pregnancy. Data on exposures are presented in Table 2. The obtained exposure profile was complete, meaning that all 16 exposure data were available, for 157 individuals. Highest variability in exposures were observed for arsenic, followed by cadmium and by MECPP as can be seen from the coefficient of variation which are 158%, 116% and 90%, respectively. Pearson correlation coefficients (Table S1) showed a high correlation between the three PCB congeners (r = 0.77–0.90), between the PCB congeners and p,p’-DDE (r = 0.46–0.54), the perfluorinated compounds (r = 0.50) and arsenic and methylmercury (r = 0.46). 

### 3.1. Single Pollutant Regression Models 

**Figure 2:**
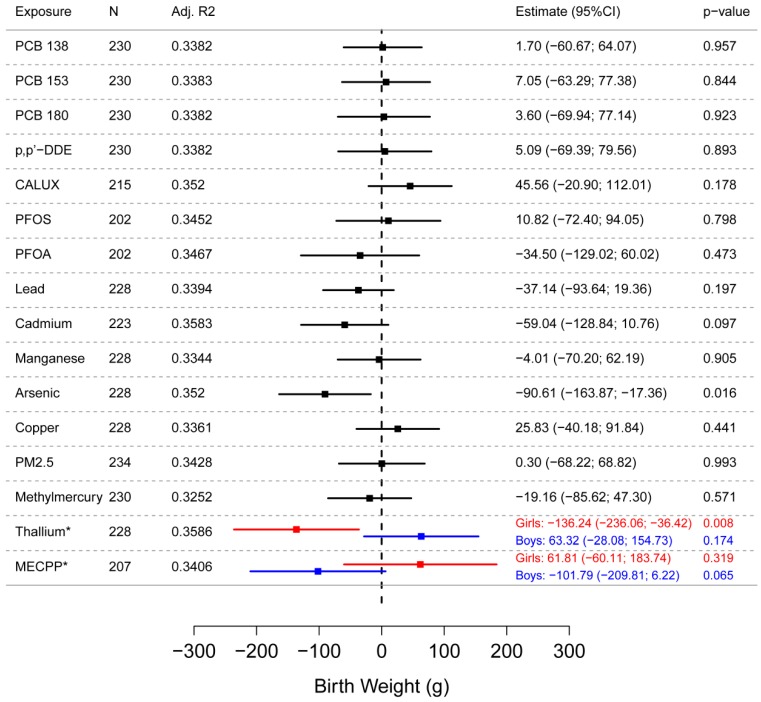
Association between maternal exposure and birth weight based on single pollutant regression models. The estimated effects (for an increase of the Z-score of the exposure biomarker with the interquartile range) and corresponding confidence limits (95%) are shown by squares and lines, respectively. Significance (at the 5% level) is demonstrated when the confidence interval does not include 0. The models are adjusted for gestational age, child’s sex, smoking of the mother during pregnancy, parity and maternal prepregnancy BMI. * For thallium and MECPP effect modification by sex was observed. A separate estimate was calculated for boys (blue) and girls (red). N = Number of samples; Adj. R^2^ = Adjusted R^2^.

Single pollutant multiple linear regression models showed a significant inverse association (*p* = 0.016) between increasing arsenic concentrations in cord blood and lower birth weight (Figure 2). For an increase of arsenic cord blood levels with the interquartile range in Z-score, birth weight decreased by 91 g (95% CI: 17; 164 g). The models were adjusted for gestational age, child’s sex, smoking of the mother during pregnancy, parity and maternal prepregnancy BMI. The model assumptions were fulfilled, and the effect estimates and significance levels of the associations did not change substantially after fitting the model without influential outliers. Except for thallium, the associations were not modified by sex when looking at the cross-product term of sex with the pollutant levels (*p* > 0.10). When including the interaction with sex in the model, thallium was negatively associated with birth weight in girls (*p* = 0.008), not in boys. The associations were not modified by smoking status when looking at the cross-product term of smoking status with the principal components. The adjusted R^2^ value when regressing birth weight in function of all explanatory variables except the exposure was equal to 0.3458. The additional fraction explained by the contaminant is very small (about 1%) (Figure 2). 

### 3.2. Principal Component Regression (PCR) 

**Figure 3:**
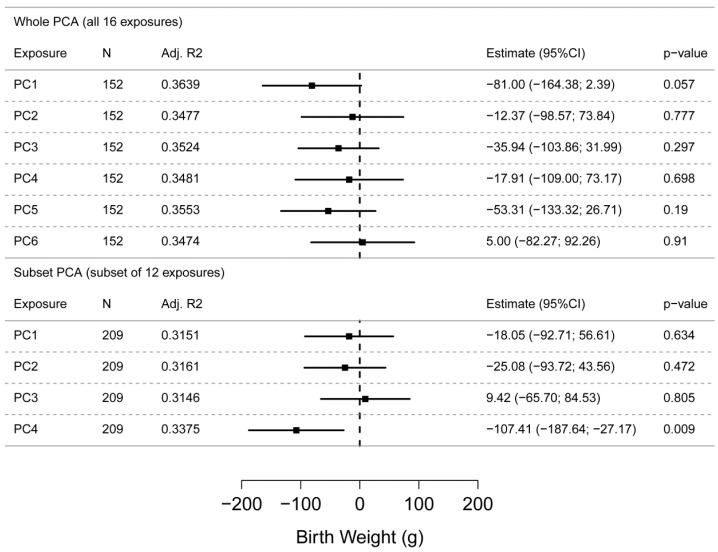
Results of the Principal Component Regression (PCR) for the whole PCA (all 16 exposures) and subset PCA (subset of 12 exposures). The estimated effects (for an increase of the Z-score of the principal component with the interquartile rang) and corresponding confidence limits (95%) are shown by squares and lines, respectively. Significance (at the 5% level) is demonstrated when the confidence interval does not include 0. The models are adjusted for gestational age, child’s sex, smoking of the mother during pregnancy, parity and maternal prepregnancy BMI. PC = Principal component; N = Number of samples; Adj. R^2^ = Adjusted R^2^.

The exposure biomarker Z-scores quantified in the present study were clustered using PCA. As PCA works on the complete set of data for all exposures, only 157 individuals could be used in this analysis (further referred to as whole PCA). As a sensitivity analysis, PCA was also performed on 12 out of the 16 exposures (excluding PFOA, PFOS, MECPP and Calux) available for 217 individuals (further referred to as subset PCA). PCA identified six and four principal components (PCs) in the exposure data with eigenvalues >1 for the whole and subset PCA, respectively. The composition of the principal components is presented in Table S2. In total, 66% and 63% of the variability in the exposure data set could be explained by the six and four PCs in the whole and subset PCA, respectively. PC1 explains, respectively, 22% and 28% of the variability in the exposure data set, and is composed merely by the different PCB congeners (PCB-183, PCB-153 and PCB-180) for the whole and subset PCA. In Figure 3 the results of principal component regression are presented, *i.e.*, it displays the associations between the principal components and birth weight, adjusted for gestational age, child’s sex, smoking of the mother during pregnancy, parity and maternal prepregnancy BMI. For the PCs composed from the whole PCA, none of the principal components were statistically significant (*p* < 0.05) associated with birth weight. However, since the models were built for a subset of 152 from 248 individuals, statistical power may be reduced. For the PCs resulting from the subset PCA, a significant negative association with birth weight was found for PC4 (*p* = 0.009) constituted by arsenic and cadmium. The associations were not modified by sex or smoking status.

### 3.3. Exploring the Effect of Mixtures 

**Figure 4:**
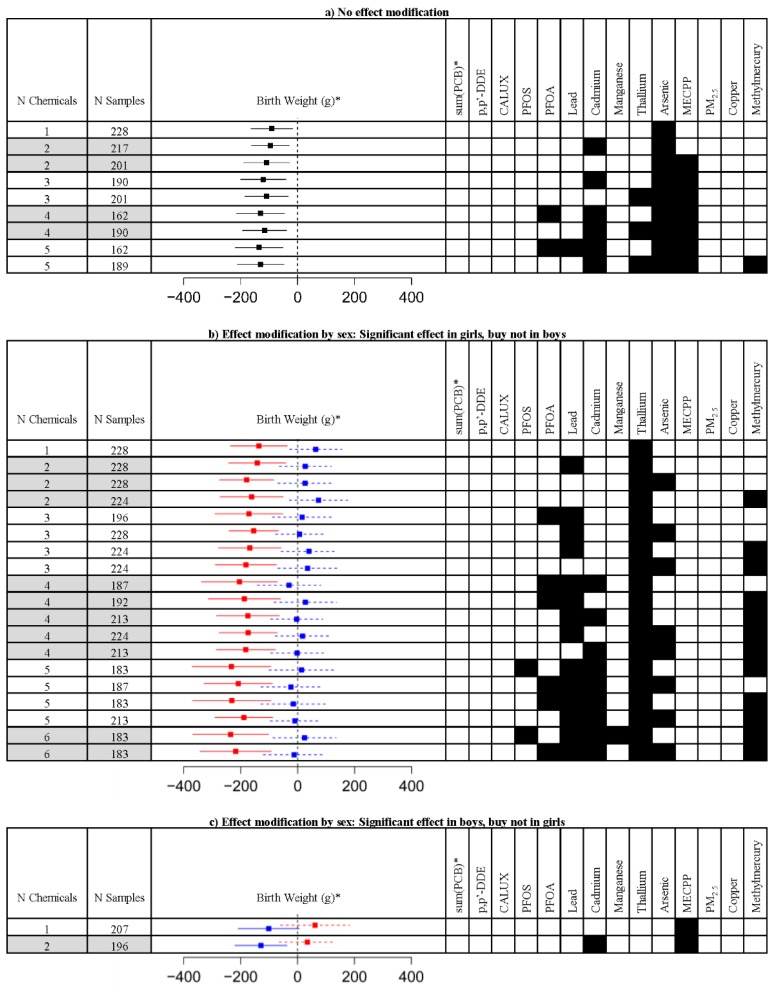
Results of mixture regression algorithm showing an increase in the association between birth weight and exposure by growing complexity of mixtures. The black rectangles indicate the exposure biomarkers that are included in the model. The estimate of the association between exposure and birth weight is given for an increase of the mixture Z-score with the interquartile range. The estimated effects and corresponding confidence limits (95%) are shown by squares and lines, respectively. Significance (at the 5% level) is demonstrated when the confidence interval does not include 0. (**a**) The models adjusted for gestational age, child’s sex, smoking of the mother during pregnancy, parity and maternal prepregnancy BMI; (**b**,**c**) Models that include modification of the effect by child’s sex that focus on significant effects in girls and boys, respectively. The effect in girls is shown in red, while the effect in boys is shown in blue. Numerical data (estimates, 95% confidence limits, *p*-value, R^2^, and MSE) are included in Tables S3–S5. * Three marker PCBs (*i.e.*, PCB-180, PCB-153, and PCB-138) were summed prior to analysis.

The effect of stepwise combinations of increasing number of chemicals was compared based on the estimate and p-value of the association between mixture Z-scores and birth weight (Figure 4, Tables S3–S5). Due to the high correlation between the PCB congeners (Pearson correlation coefficients between 0.77 and 0.9), the sum of three marker PCBs (*i.e.*, PCB-180, PCB-153, and PCB-138) was z-transformed as a measure for PCB exposure in the approach exploring mixtures instead of considering the congeners separately. As for the separate PCBs, the sum of marker PCBs was not significantly associated with birth weight (*p* = 0.92). The models were adjusted for gestational age, child’s sex, smoking of the mother during pregnancy, parity and maternal prepregnancy BMI. When effect modification by sex was not assumed (Figure 4a), mixtures with arsenic, cadmium and MECPP showed a strong association with birth weight as can be derived from the estimates. The mixtures with the highest association with birth weight were composed of five chemicals, *i.e.*, PFOA, lead, cadmium, arsenic, and MECPP (estimate of −135 g for an increase in IQR of the average Z-score, *p* = 0.0019); and cadmium, thallium, arsenic, MECPP, and methylmercury (estimate = −130 g, *p* = 0.0021).

Including effect modification by sex in the regression models, the effects of mixtures in girls (Figure 4b) were stronger as compared to boys (Figure 4c). Overall, thallium and MECPP were the main compounds that showed different effects in girls and boys, respectively. A strong effect of mixture exposures in girls was identified when combining six chemicals, *i.e.*, PFOS, lead, cadmium, manganese, thallium, and methylmercury (estimate of −235 g for an increase in IQR of the average Z-score, *p* = 0.0006); and PFOA, lead, cadmium, thallium, arsenic, and methylmercury (estimate = −218 g, *p* = 0.0007). In boys, the mixture of MECPP with cadmium (estimate = −129 g, *p* = 0.0061) and showed a stronger association with birth weight as compared to MECPP alone (estimate = −101 g, *p* = 0.06).

## 4. Discussion

In 248 mother-infant pairs characterized by 16 exposure parameters, only arsenic exposure measured in cord blood related significantly with birth weight in a single pollutant model adjusted for confounders and significant covariates. This was seen irrespective of gender and was described earlier and linked to inhibition of placental angiogenesis by arsenic [29]. Potential sources of As exposure include food, such as rice and fish [56,57]. The concentrations in drinking water in Belgium are well controlled, however local well water may contain higher levels. Remy *et al.* [29] summarized the current evidence of elevated risk of adverse birth outcomes related with *in utero* or early-life exposure to As. Most studies focus on areas with high As concentrations in drinking water, far above 40 µg/L and exceeding the current WHO drinking water guidelines of 10 µg/L. Although epidemiologic reports that focus on the low exposure range are scarce, low exposure levels have been associated with reduced birth weight, birth length, and chest and head circumference before [58,59]. In Flanders, a region in Belgium with even 10 times lower As concentrations as compared to both studies mentioned above, the inverse association could be confirmed. Thallium measured in cord blood samples associated also significantly with decreased birth weight, but this was only manifest in girls. These findings add to a recent report from a Chinese birth cohort that linked maternal urinary thallium levels with low birth weight in a case control approach but this result was not specific for girls [60].

We applied in this study two different statistical models for testing the effects of combined exposures on birth weight as this exposure information is available in our cohort. One of the strengths of this study is that the selected statistical models were able to adjust for potential confounders. It is well known that variables such as sex, smoking, gestational age, prepregnancy BMI of the mother, and parity may have an influence on birth outcome [61,62,63]. Models that combined exposures and were adjusted for the confounders and significant covariates provide new insight in the negative association between chemical exposures and birth weight.

Principal component analysis allowed identifying six mixtures that explained together 66% of the variability in exposure, but regression analysis did not show a significant association with birth weight. This may be partially due to the missing values for some of the exposures or confounders reducing statistical power (only 152 complete cases). As a sensitivity analysis, the PCA was constructed for a subset of exposures yielding a higher sample size and in the same time retaining as much as possible exposures. The resulting principal component constituted by cadmium and arsenic was negatively associated with birth weight (209 complete cases) (*p* = 0.009). The negative association of maternal cadmium levels with birth weight was also found in the EDEN mother–child cohort study [64].

Another limitation of PCA was that the factors that were produced reflect only the exposure variability and do not take into account already known relationships of chemicals with the effect. The strength of the principal component analysis is being able to deal with multicollinearity by the construction of a limited number of exposure factors that may include many individual exposure parameters. However, interactions between chemicals (synergism and antagonism) are not considered. A recently published study examined the effect of mixed prenatal exposures to potential obesogenic chemicals on overweight measured at Age 7 applying PCA regression [23]. This study identified an organochlorine factor that was positively associated with BMI Z-scores and with overweight while a phthalate factor was inversely associated with overweight. 

Exploring the effect of mixtures, we build an algorithm to determine whether the estimated effect estimate of more complex mixtures on birth weight is more significant compared to mixtures composed of a smaller set of chemicals. The most significant associations, as derived from single pollutant associations, were selected as starting point (arsenic in both sexes, thallium in girls, MECPP in boys). By an iterative approach more and more exposure parameters were included while the strength and significance of the association was evaluated. If both sexes were included in the analysis, cadmium, lead, PFOA, and MECPP enhanced the association with birth weight compared to arsenic alone and the significance increased. Also mercury and thallium contributed to mixtures that significantly reduced birth weight. Current literature on the associations between PFOA and PFOS and human fetal growth has been recently reviewed [65]. Based on 14 publications, it was concluded that PFOA and PFOS exposures in pregnancy were associated with lower average birth weights in human newborns in most studies, although not all results were statistically significant. Negative effects of maternal lead levels on birth weight have been reported in the ALSPAC cohort study [66].

Models that included effect modification by sex, demonstrated that mixtures containing thallium and five other exposure markers (PFOS, lead, cadmium, manganese, and mercury) were 2.6 times stronger in reducing birth weight in girls than if arsenic alone was included in the analysis of the full cohort. In boys, the mixture of MECPP and Cd showed stronger associations with birth weight compared to MECPP alone. Our results confirm the sex specific effects of chemical exposures on birth outcome parameters as demonstrated in several studies and which has been linked to the endocrine disrupting effects of chemicals that act differently in developing males and females [67]. 

In this study we focused on birth weight as a continuous outcome parameter. In the single pollutant model a decline in birth weight with about 90 g was found for an increase in As with the IQR, while in models considering mixtures an effect estimate of 235 g was found in girls for an IQR increase for a certain mixture. It is difficult to assess public health implication for a reduction in birth weight with several hundred grams. For this concern, it would be interesting to investigate the association of (mixtures of) pollutants with adverse birth outcomes, like low birth weight (<2500 g) and small for gestational age (SGA). In our study, only five and 17 babies were found with low birth weight and SGA, respectively. This makes the power of the hypothesis very small and the probability of separation of data points very likely. Future research on adverse health outcomes should be performed. However, relative risk estimates of later disease development associated with birth weight changes have been published in the context of type 2 diabetes [68]. 

Although our approach for analyzing the effects of combined chemical exposures should be further refined, as a proof of concept we found that including different exposures strengthened the negative association of chemical exposure with birth weight. Whether this is due to independent action of the chemicals or to interactions between the chemicals could not be identified in our analysis. Future statistical models may benefit from taking advantage or including existing information on the mode of action of chemicals to construct mechanistically based exposure factors. Nevertheless the fraction explained by either a single pollutant or a mixture of pollutants is small compared to the fraction explained by the explanatory variables gestational age, child’s sex, smoking of the mother during pregnancy, parity and maternal prepregnancy BMI. However, the findings demonstrated that aside from known birth weight influencing parameters, also prenatal environmental exposure to a mixture of pollutants, may contribute significantly to changes in birth weight.

Another limitation to identify exposures that have an important association with birth weight is related to the exposure gradient of the different chemicals in the cohort. If the exposure gradient is small as for example for PM exposures in our cohort, much larger cohorts are needed to identify significant effects. Arsenic, cadmium and MECPP showed the largest variability in exposure in our cohort. Not only the strength of the dose-response relation, but also the existing exposure gradient influences the estimate of the association. Exposure gradients are cohort specific, this may explain the inconsistency in results, which is frequently encountered in observational dose effect studies. With regards to arsenic it should be mentioned that different arsenic species may differ in toxicity. However, speciation analysis was not available in the cord blood samples. Although maternal urinary As concentrations were not analyzed in our cohort, we have urinary levels of total As and toxicologically relevant As (the sum of inorganic As, monomethylarsonic acid (MMA), and dimethylarsinic acid (DMA)) in a cohort from 101 women aged between 20 and 40 sampled in the same campaign. In this population, which is representative for Flanders, toxicologically relevant As was about 25% of total arsenic in urinary samples. Total As and toxicologically relevant As were highly correlated (Pearson correlation coefficient = 0.62; *p*-value = 1.58E-12). The median levels of total As and toxicological relevant As were 16.2 µg/L (25th percentile: 6.9 µg/L; 75th percentile: 30.1 µg/L) and 4.3 µg/L (25th percentile: 2.9 µg/L; 75th percentile: 7.7 µg/L), respectively. 

Combining different exposures is already closer to real life situations than analyzing exposures effects from pollutants one by one. We should however keep in mind that although human biomonitoring provides direct information as to what chemicals and how much are being absorbed into the body, it is generally a one-time snapshot of an individual's exposure. The accuracy of this snapshot depends on the chemical of interest, its half-life in the body, its target, the sensitive exposure window and the analytical accuracy. Although we measured markers of 16 compounds, we did not yet get the complete picture of exposures and confounders. 

We explored a novel approach on our dataset by using the average of the z-scores of the components constituting a mixture as the predictor variable of birth weight in a regression model to obtain the estimated effect of mixture exposure on birth weight. All possible mixtures were compared. However, the approach has some limitations. For the interpretation of the results, one must be aware that this average z-score cannot reflect the relative toxic potency or interaction of the selected exposures in the mixture and that collinearity between the selected exposures is not fully taken into account in this measure of “total exposure”. Due to the high correlation between the PCB congeners (Pearson correlation coefficients between 0.77 and 0.9) the sum of three marker PCBs (*i.e.*, PCB-180, PCB-153, and PCB-138) was z-transformed as a measure for PCB exposure in the approach exploring mixtures instead of considering the congeners separately. This avoids that the average z-score is predominantly determined by PCBs when two or more PCBs are in the mixture together with other compounds. Correlation coefficients for all other pairs were below 0.5 (except for the correlation between PCB-153 and DDE (r = 0.54)). As such, we decided to enter the z-scores of the other chemicals exposure markers as separate entities in the analysis. 

To enhance comparability between the models, the estimated effect on birth weight was expressed for an increase in average z-score with the IQR. If the regression coefficient increased by adding an extra chemical, the estimated effect on birth weight was considered to be elevated. However, we cannot exclude that some bias was introduced by correlated exposures. Averaging z-scores of all possible combinations of exposures and algorithm based model building is a novel approach to identify exposures that contribute to the health effect. However, more research is needed to enhance the approach to solve its current limitations.

## 5. Conclusions

All the significant associations that were observed in our study between prenatal chemical exposures and birth weight were negative associations, irrespective of the statistical models that were used. Of the 16 exposure parameters that were studied by single pollutant regression models, only cord arsenic levels showed a statistically significant negative association with birth weight. When considering effect modification by sex, exposure to thallium reduced birth weight significantly only in girls. Principal component regression with cadmium and arsenic as the main contributors to the principal component showed a negative association with birth weight. Exploring mixtures by a novel approach showed that the negative association of exposures with birth weight gained strength if up to five exposure parameters were included in the models. Combined exposure to arsenic, cadmium, lead, PFOA, and MECPP enhanced the association with birth weight compared to exposure to arsenic alone. Effects were even more pronounced if sex modification was considered. Effects in girls were enhanced, with the highest association for mixtures containing thallium, PFOS, lead, cadmium, and manganese. Lower birth weight is a risk factor for diseases later in life. Our analysis highlights the importance of preventing prenatal chemical exposure at the start of life.

## Figures and Tables

**Table 1:** Characteristics of newborns and their mothers among 248 participants.

Continuous Parameters	N	N Missing	Median (min-max)
Birth weight (g)	248	0	3540 (2175–4950)
Gestational Age (weeks)	243	5	40 (34–42)
Categorical Parameters	Class	N	Percentage
Child gender	Boy	128	51.6%
	Girl	120	48.4%
	Missing	0	0.0%
Maternal age at delivery (years)	≤25	27	10.9%
	(25,30)	92	37.1%
	(30,35)	94	37.9%
	>35	35	14.1%
	Missing	0	0.0%
Maternal pre-pregnancy BMI (kg/m^2^)	<18.5	15	6.0%
	(18.5,25)	172	69.4%
	(25,30)	37	14.9%
	≥30	22	8.9%
	Missing	2	0.8%
Maternal height (cm)	<164	62	25.0%
	(164,168)	58	23.4%
	(168,171)	56	22.6%
	≥171	70	28.2%
	Missing	2	0.8%
Parity	0	99	39.9%
	1	82	33.1%
	≥2	66	26.6%
	Missing	1	0.4%
Caesarean section	Yes	12	4.8%
	No	235	94.8%
	Missing	1	0.4%
Maternal education	Lower secondary education	22	8.9%
	Higher secondary education	74	29.8%
	Higher education	149	60.1%
	Missing	3	1.2%
Use of folic acid during pregnancy	Yes	157	63.3%
	No	91	36.7%
	Missing	0	0.0%
Infections/complications during pregnancy	No	151	60.9%
	Yes	93	37.5%
	Missing	4	1.6%
Maternal smoking during pregnancy	Yes	29	11.7%
	No	213	85.9%
	Missing	6	2.4%
Maternal smoking prior to pregnancy	Never smoked	135	54.4%
	Ex-smoker	37	14.9%
	Less than daily	16	6.5%
	Daily	56	22.6%
	Missing	4	1.6%
Maternal alcohol use before pregnancy	Never	36	14.5%
	Less than monthly	60	24.2%
	Less than weekly	56	22.6%
	Weekly	95	38.3%
	Missing	1	0.4%
Maternal alcohol use during pregnancy	Yes	104	41.9%
	No	142	57.3%
	Missing	2	0.8%
Stress during pregnancy	Never-sometimes a little	180	72.6%
	Usually a little-always	65	26.2%
	Missing	3	1.2%
Pressure during pregnancy	Never-sometimes a little	134	54.0%
	Usually a little-always	112	45.2%
	Missing	2	0.8%

**Table 2:** Exposure to environmental chemicals.

Exposure marker	Matrix	N	LOD/LOQ	N < LOD/LOQ (%)	Geomean (95% CI)	P25–P75
Arsenic (µg/L)	Cord blood	242	LOD = 0.028 µg/L	1 (0.4%)	0.561 (0.485–0.648)	0.256–1.223
Cadmium (µg/L)	Maternal blood	237	LOD = 0.06 µg/L	1 (0.4%)	0.316 (0.291–0.344)	0.210–0.434
Copper (µg/L)	Cord blood	242	LOD = 2.04µg/L	0 (0%)	598 (584–613)	534–679
Dichlorodiphenyldichloroethylene (ng/g lipids)	Cord plasma	243	LOQ = 20 ng/L	0 (0%)	77.9 (71.3–85.2)	47.1–126.0
Dioxin-like compounds (pg Calux TEQ/g lipids)	Cord plasma	227	LOD = 9.7 pg Calux TEQ/g lipids	14 (6%)	17.4 (16.3–18.6)	13.0–24.0
Lead (µg/L)	Cord blood	242	LOD = 1.9 µg/L	0 (0%)	8.64 (8.08–9.23)	6.52–11.38
Manganese (µg/L)	Cord blood	242	LOD = 0.86 µg/L	0 (0%)	30.9 (29.5–32.4)	24.6–38.9
Methylmercury (µg/g)	Maternal hair	244	LOD = 0.00004 µg/g	0 (0%)	0.255 (0.230–0.283)	0.161–0.441
Mono-(2-ethyl-5-carboxypentyl) phthalate (µg/L)	Cord plasma	219	LOQ = 0.07–0.18 µg/L	0 (0%)	0.699 (0.628–0.779)	0.380–1.300
Particulate matter (≤2.5 µM) (µg/m³)	RIO model	242	/	/	19.6 (19.3–19.9]	18.4–21.4
Perfluorooctane sulfonate (µg/L)	Cord plasma	213	LOD = 0.3 µg/L	0 (0%)	2.63 (2.45–2.83)	1.70–3.80
Perfluorooctanoic acid (µg/L)	Cord plasma	213	LOD = 0.3 µg/L	0 (0%)	1.52 (1.44–1.61)	1.10–2.10
Polychlorinated biphenyl-138 (ng/g lipids)	Cord plasma	243	LOQ = 20 ng/L	41 (17%)	16.4 (15.1–17.9)	12.0–25.9
Polychlorinated biphenyl-153 (ng/g lipids)	Cord plasma	243	LOQ = 20 ng/L	8 (3%)	26.4 (24.5–28.5)	18.4–39.3
Polychlorinated biphenyl-180 (ng/g lipids)	Cord plasma	243	LOQ = 20 ng/L	50 (21%)	14.6 (13.4–15.9)	9.40–23.11
Thallium (µg/L)	Cord blood	242	LOD = 0.001 µg/L	0 (0%)	0.017 (0.016–0.018)	0.014–0.021

Notes: Abbreviations: N: Number of participants; P25: 25th percentile; P75: 75th percentile; 95% CI: 95% confidence interval; LOD: Limit of detection; LOQ: Limit of quantification; Geomean: geometric mean.

## Supplementary Materials

The following are available online at www.mdpi.com/1660-4601/13/5/495/s1. Table S1: Pearson correlation matrix of the exposure data. Table S2: Results of the Principal Component Analysis (PCA): component loading weights for the exposures. Table S3: Mixture regression models with significant negative associations between maternal exposure and birth weight. Table S4: Mixture regression models with significant negative associations between maternal exposure and birth weight in girls but not in boys. Table S5: Mixture regression models with significant negative associations between maternal exposure and birth weight in boys but not in girls
