# Supplementary material for: Combined Effects of Prenatal Exposures to Environmental Chemicals on Birth Weight
Source: Int J Environ Res Public Health. 2016 May 12;13(5):495. doi: 10.3390/ijerph13050495 (PMC4881120; doi:10.3390/ijerph13050495)
Supplement: Supplementary file 1 [file ijerph-13-00495-s001.pdf]

Supplementary Materials: Combined Effects of Prenatal Exposures to Environmental Chemicals on Birth Weight

Eva Govarts, Sylvie Remy, Liesbeth Bruckers, Elly Den Hond, Isabelle Sioen, Vera Nelen, Willy Baeyens, Tim S Nawrot, Ilse Loots, Nick Van Larebeke and Greet Schoeters

Table S1. Pearson correlation matrix of the exposure data.

	PCB 180	PCB 153	PCB 138	p,p′-DDE	CALUX	PFOS	PFOA	Lead	Cadmium	Manganese	Thallium	Arsenic	MECPP	$PM_{2.5}$	Copper	Methylmercury
**PCB 180**	1; 1															
**PCB 153**	0.9; <0.001	1; 1														
**PCB 138**	0.77; <0.001	0.8; <0.001	1; 1													
**p,p′-DDE**	0.46; <0.001	0.54; <0.001	0.48; <0.001	1; 1												
**CALUX**	0.21; 0.002	0.15; 0.022	0.1; 0.152	0.09; 0.155	1; 1											
**PFOS**	0.13; 0.053	0.22; 0.001	0.14; 0.045	0.2; 0.004	0.02; 0.816	1; 1										
**PFOA**	0.16; 0.018	0.19; 0.005	0.06; 0.414	0.07; 0.307	0.09; 0.205	0.5; <0.001	1; 1									
**Lead**	0.24; <0.001	0.24; <0.001	0.17; 0.011	0.22; <0.001	−0.02; 0.777	0.1; 0.159	0.16; 0.019	1; 1								
**Cadmium**	0.17; 0.01	0.11; 0.085	0.09; 0.156	0.01; 0.901	0.1; 0.164	−0.17; 0.014	0.03; 0.718	0.14; 0.035	1; 1							
**Manganese**	0.13; 0.053	0.08; 0.203	0.15; 0.023	−0.06; 0.326	−0.09; 0.172	−0.2; 0.003	−0.09; 0.221	0.2; 0.002	-0.03; 0.62	1; 1						
**Thallium**	−0.11; 0.097	−0.09; 0.163	−0.14; 0.036	−0.03; 0.624	−0.09; 0.196	0.02; 0.795	0.07; 0.291	0.13; 0.047	0.03; 0.659	0.14; 0.026	1; 1					
**Arsenic**	0.26; <0.001	0.27; <0.001	0.17; 0.01	0.03; 0.7	0.05; 0.503	0.09; 0.216	0.1; 0.134	0.21; 0.001	−0.05; 0.492	0.15; 0.02	0.05; 0.446	1; 1				
**MECPP**	−0.03; 0.617	−0.07; 0.343	−0.09; 0.209	0.01; 0.859	−0.02; 0.827	0.07; 0.315	0.11; 0.126	0.01; 0.884	-0.08; 0.238	−0.07; 0.34	0.13; 0.053	−0.07; 0.328	1; 1			
**$PM_{2.5}$**	0.19; 0.003	0.16; 0.017	0.21; <0.001	0.09; 0.15	0.18; 0.008	0.07; 0.339	0.07; 0.29	0.05; 0.48	0.17; 0.01	−0.01; 0.921	−0.06; 0.394	−0.05; 0.455	−0.15; 0.028	1; 1		
**Copper**	0.06; 0.339	0.02; 0.818	0.03; 0.659	0.01; 0.821	−0.1; 0.138	−0.1; 0.133	−0.07; 0.351	0.15; 0.018	−0.04; 0.553	0.38; <0.001	0.29; <0.001	0.08; 0.192	0.2; 0.004	−0.11; 0.102	1; 1	
**Methyl-mercury**	0.29; <0.001	0.3; <0.001	0.15; 0.018	0.11; 0.094	0.07; 0.319	0.35; <0.001	0.23; 0.001	0.15; 0.019	−0.09; 0.176	0.11; 0.082	0.02; 0.786	0.46; <0.001	−0.01; 0.845	−0.05; 0.46	0.06; 0.369	1; 1

Note: Pearson correlation coefficient followed by corresponding *p*-value.

Table S2. Results of the Principal Component Analysis (PCA): component loading weights for the exposures.

**Exposure**	**Whole PCA (All 16 Exposures)**						**Subset PCA (Subset of 12 Exposures)**			
**Principal Component**	**PC1**	**PC2**	**PC3**	**PC4**	**PC5**	**PC6**	**PC1**	**PC2**	**PC3**	**PC4**
**Explained variance (%)**	**22%**	**11%**	**10%**	**8%**	**8%**	**6%**	**28%**	**15%**	**11%**	**9%**
PCB 138	**0.80**	0.08	−0.27	0.02	−0.26	−0.09	**0.85**	−0.20	0.07	−0.17
PCB 153	**0.92**	0.04	−0.19	0.03	−0.11	0.03	**0.93**	−0.12	−0.03	−0.07
PCB 180	**0.88**	0.08	−0.17	0.05	−0.05	0.13	**0.92**	−0.09	0.01	−0.03
p,p′-DDE	**0.56**	−0.06	−0.05	0.15	−0.39	0.02	**0.62**	−0.19	0.09	−0.33
CALUX	0.23	−0.27	−0.15	0.16	0.27	**0.65**	/	/	/	/
PFOS	0.40	**−0.57**	**0.43**	−0.11	−0.08	−0.28	/	/	/	/
PFOA	0.31	**−0.42**	**0.52**	0.16	0.21	−0.14	/	/	/	/
Lead	**0.41**	0.27	0.27	0.30	0.16	−0.35	0.37	0.33	0.24	0.31
Cadmium	0.10	0.13	−0.08	**0.64**	**0.54**	0.12	0.15	−0.19	**0.50**	**0.63**
Manganese	0.13	**0.70**	−0.07	−0.07	−0.01	−0.20	0.19	**0.57**	0.29	−0.12
Thallium	−0.07	0.34	**0.57**	0.29	0.02	0.01	−0.09	**0.51**	**0.41**	0.01
Arsenic	**0.47**	0.25	0.13	−0.36	**0.48**	0.06	0.37	**0.46**	**−0.45**	**0.43**
Copper	0.05	**0.57**	0.36	0.15	−0.25	0.20	0.10	**0.64**	0.38	−0.31
MECPP	0.01	−0.12	**0.49**	0.17	**−0.45**	**0.41**	/	/	/	/
$PM_{2.5}$	0.10	−0.35	−0.25	**0.47**	0.05	−0.27	0.20	−0.38	0.37	0.27
Methylmercury	**0.50**	−0.03	0.31	**−0.52**	0.29	0.11	**0.40**	**0.40**	**−0.56**	0.19

Abbreviations: PC: Principal Component.

Table S3. Mixture regression models with significant negative associations between maternal exposure and birth weight.

N Exposures	*p*-Value	Estimate (95% CI) *	R^2 Adj.	MSE	N	sum(PCB) *	p,p′-DDE	CALUX	PFOS	PFOA	Lead	Cadmium	Manganese	Thallium	Arsenic	MECPP	$PM_{2.5}$	Copper	Methylmercury
1	0.0156	−90.59 (−163.83; −17.35)	0.35	148,758	228										■				
2	0.0045	−95.92 (−161.77; −30.08)	0.37	145,897	217							■			■				
2	0.0083	−108.69 (−189.04; −28.33)	0.34	138,750	201										■	■			
3	0.0030	−120.97 (−200.37; −41.57)	0.36	135,197	190							■			■	■			
3	0.0054	−108.84 (−185.19; −32.49)	0.35	138,201	201									■	■	■			
4	0.0025	−130.32 (−214.13; −46.51)	0.38	127,503	162					■		■			■	■			
4	0.0036	−115.78 (−193.16; −38.4)	0.36	135,427	190							■		■	■	■			
5	0.0019	−135.14 (−219.7; −50.59)	0.38	127,083	162					■	■	■			■	■			
5	0.0021	−129.75 (−211.89; −47.61)	0.36	134,989	189							■		■	■	■			■

Notes: * The estimate is calculated for an increase of the mixture Z-score with the interquartile range. The model is adjusted for gestational age, child’s sex, smoking of the mother during pregnancy, parity and maternal prepregnancy BMI. The black rectangles indicate the exposure biomarkers that are included in the model. MSE: Mean squared error. * 3 marker PCB’s (*i.e.*, PCB 180, PCB 153, and PCB 138) were summed prior to analysis.

Table S4. Mixture regression models with significant negative associations between maternal exposure and birth weight in girls but not in boys.

N Chemicals	$p_{Interaction}$	p_{Girls}	Estimate (95% CI)$_{Girls}$ *	p_{Boys}	Estimate (95% CI)$_{Boys}$ *	R^2Adj.	MSE	N Samples	Sum(PCB) *	p,p′-DDE	CALUX	PFOS	PFOA	Lead	Cadmium	Manganese	Thallium	Arsenic	MECPP	$PM_{2.5}$	Copper	Methylmercury
1	0.0037	**0.0077**	**−136.22 (−236.02; −36.42)**	0.1735	63.32 (−28.07; 154.7)	0.36	147,243	228									■					
2	0.0140	**0.0058**	**−141.88 (−242.27; −41.49)**	0.5634	26.73 (−64.32; 117.78)	0.36	147,950	228						■			■					
2	0.0027	**0.0003**	**−179.08 (−274.18; −83.99)**	0.5888	26.27 (−69.37; 121.9)	0.38	144,119	228									■	■				
2	0.0022	**0.0044**	**−161.56 (−272.26; −50.86)**	0.1609	73.09 (−29.32; 175.49)	0.35	147,443	224									■					■
3	0.0192	**0.0053**	**−170.94 (−290.38; −51.5)**	0.7685	15.6 (−88.8; 120)	0.36	143,296	196					■	■			■					
3	0.0087	**0.0005**	**−154.45 (−240.96; −67.93)**	0.8782	6.54 (−77.49; 90.57)	0.37	145,208	228						■			■	■				
3	0.0055	**0.0028**	**−168.15 (−277.79; −58.5)**	0.4252	39.74 (−58.29; 137.77)	0.34	147,847	224						■			■					■
3	0.0040	**0.0010**	**−181.7 (−288.8; −74.6)**	0.5113	34.74 (−69.33; 138.81)	0.35	146,567	224									■	■				■
4	0.0398	**0.0029**	**−204.37 (−337.69; −71.05)**	0.5904	−30.42 (−141.76; 80.92)	0.37	142,594	187					■	■	■		■					
4	0.0114	**0.0039**	**−187.3 (−313.72; −60.87)**	0.6287	26.97 (−82.88; 136.82)	0.34	143,733	192					■	■			■					■
4	0.0171	**0.0021**	**−174.83 (−285.2; −64.46)**	0.9393	−3.53 (−94.79; 87.73)	0.35	146,637	213						■	■		■					■
4	0.0071	**0.0009**	**−173.85 (−275.46; −72.25)**	0.7247	17.16 (−78.76; 113.09)	0.35	146,720	224						■			■	■				■
4	0.0099	**0.0006**	**−182.25 (−285.82; −78.68)**	0.9627	−2.2 (−94.83; 90.43)	0.36	145,070	213							■		■	■				■
5	0.0068	**0.0010**	**−232.62 (−370.35; −94.89)**	0.8123	13.78 (−100.54; 128.09)	0.36	142,128	183				■		■	■		■					■
5	0.0203	**0.0007**	**−208.62 (−328.58; −88.66)**	0.6548	−23.79 (−128.65; 81.06)	0.38	140,587	187					■	■	■		■	■				
5	0.0153	**0.0011**	**−231.32 (−368.97; −93.66)**	0.7930	−15.15 (−128.96; 98.65)	0.36	142,274	183					■	■	■		■					■
5	0.0078	**0.0003**	**−188.92 (−290.39; −87.44)**	0.8248	−9.72 (−96.22; 76.77)	0.36	144,098	213						■	■		■	■				■

Table S4. *Cont.*

N Chemicals	$p_{Interaction}$	p_{Girls}	Estimate (95% CI)$_{Girls}$ *	p_{Boys}	Estimate (95% CI)$_{Boys}$ *	R^2Adj.	MSE	N Samples	Sum(PCB) *	p,p′-DDE	CALUX	PFOS	PFOA	Lead	Cadmium	Manganese	Thallium	Arsenic	MECPP	$PM_{2.5}$	Copper	Methylmercury
6	0.0032	**0.0006**	**−235.45 (−368.62; −102.29)**	0.6698	24 (−86.89; 134.88)	0.36	141,171	183				■		■	■	■	■					■
6	0.0140	**0.0007**	**−217.71 (−342.6; −92.83)**	0.8211	−12.43 (−120.77; 95.91)	0.36	141,619	183					■	■	■		■	■				■

Notes: * The estimate is calculated for an increase of the mixture Z-score with the interquartile range. The model is adjusted for gestational age, smoking of the mother during pregnancy, parity and maternal prepregnancy BMI. The black rectangles indicate the exposure biomarkers that are included in the model. MSE: Mean squared error. * 3 marker PCB's (*i.e.*, PCB 180, PCB 153, and PCB 138) were summed prior to analysis.

Table S5. Mixture regression models with significant negative associations between maternal exposure and birth weight in boys but not in girls.

N Chemicals	$p_{Interaction}$	p_{Girls}	Estimate (95% CI)$_{Girls}$ *	p_{Boys}	Estimate (95% CI)$_{Boys}$ *	R^2Adj.	MSE	N Samples	sum(PCB) *	p,p′-DDE	CALUX	PFOS	PFOA	Lead	Cadmium	Manganese	Thallium	Arsenic	MECPP	$PM_{2.5}$	Copper	Methylmercury
1	0.0458	0.3186	61.47 (−59.77; 82.71)	**0.0646**	**−101.22 (−208.64; 6.19)**	0.34	141126	207											■			
2	0.0145	0.4902	34.82 (−64.55; 134.2)	**0.0061**	**−128.65 (−220.05; −37.25)**	0.37	136225	196							■				■			

Notes: * The estimate is calculated for an increase of the mixture Z-score with the interquartile range. The model is adjusted for gestational age, smoking of the mother during pregnancy, parity, and maternal prepregnancy BMI. The black rectangles indicate the exposure biomarkers that are included in the model. MSE: Mean squared error. * 3 marker PCB's (*i.e.*, PCB 180, PCB 153, and PCB 138) were summed prior to analysis.

© 2016 by the authors; licensee MDPI, Basel, Switzerland. This article is an open access article distributed under the terms and conditions of the Creative Commons by Attribution (CC-BY) license (http://creativecommons.org/licenses/by/4.0/).
